# RP-HPLC Method Development and Validation for Determination of Eptifibatide Acetate in Bulk Drug Substance and Pharmaceutical Dosage Forms

**Published:** 2017

**Authors:** Maryam Bavand Savadkouhi, Hossein Vahidi, Abdul Majid Ayatollahi, Shirin Hooshfar, Farzad Kobarfard

**Affiliations:** a *Department of pharmaceutical biotechnology, Faculty of pharmacy, Shahid Beheshti University of medical sciences, Tehran, Iran. *; b *Students research committee, Faculty of pharmacy, Shahid Beheshti University of medical sciences, Tehran, Iran. *; c *Phytochemistry Research Center, Shahid Beheshti University of Medical Sciences, Tehran, Iran.*; d *Department of Pharmacognosy, School of Pharmacy, Shahid Beheshti University of Medical Sciences, Tehran, Iran.*; e *Department of Pharmaceutics, School of Pharmacy, Shahid Beheshti University of Medical Sciences, Tehran, Iran.*; f *Department of Medicinal Chemistry, School of Pharmacy, Shahid Beheshti University of Medical Sciences, Tehran, Iran. *; g *Central Research Laboratories, Shahid Beheshti University of Medical Sciences, Tehran, Iran.*

**Keywords:** Eptifibatide acetate, HPLC-UV, Determination, Drug substance, Formulation

## Abstract

A new, rapid, economical and isocratic reverse phase high performance liquid chromatography (RP-HPLC) method was developed for the determination of eptifibatide acetate, a small synthetic antiplatelet peptide, in bulk drug substance and pharmaceutical dosage forms. The developed method was validated as per of ICH guidelines. The chromatographic separation was achieved isocratically on C18 column (150 x 4.60 mm i.d., 5 µM particle size) at ambient temperature using acetonitrile (ACN), water and trifluoroacetic acid (TFA) as mobile phase at flow rate of 1 mL/min and UV detection at 275 nm. Eptifibatide acetate exhibited linearity over the concentration range of 0.15-2 mg/mL (r^2^=0.997) with limit of detection of 0.15 mg/mL The accuracy of the method was 96.4-103.8%. The intra-day and inter-day precision were between 0.052% and 0.598%, respectively. The present successfully validated method with excellent selectivity, linearity, sensitivity, precision and accuracy was applicable for the assay of eptifibatide acetate in bulk drug substance and pharmaceutical dosage forms.

## Introduction

**Figure 1:**
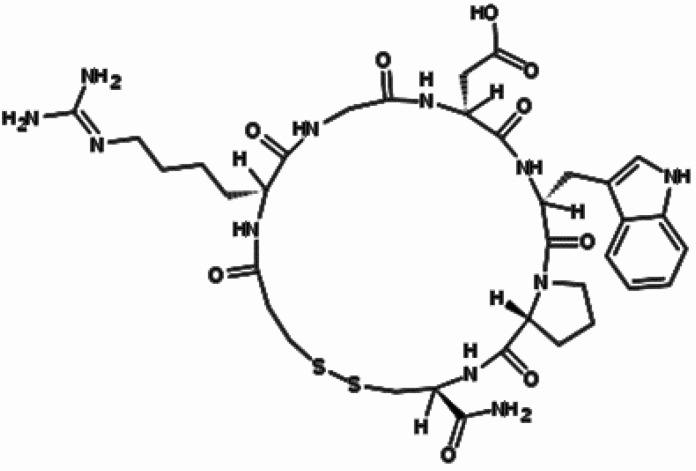
Chemical structure of eptifibatide

The cyclic heptapeptide eptifibatide (N6-(aminoiminomethyl)-N2-(3-mercapto-1-oxopropyl)-Llysylglycyl-L-α-aspartyl-L-tryptophyl-L-prolyl-L-cysteinamide,cyclic (1→6)-disulfide) (Figure 1), is a reversible inhibitor of platelet aggregation. This potent and specific glycoprotein IIb/IIIa receptor antagonist mainly indicated for treatment of acute coronary syndrome including unstable angina or non-Q-wave myocardial infarct and patients undergoing primary percutaneous coronary intervention (1-6).

**Figure 2:**
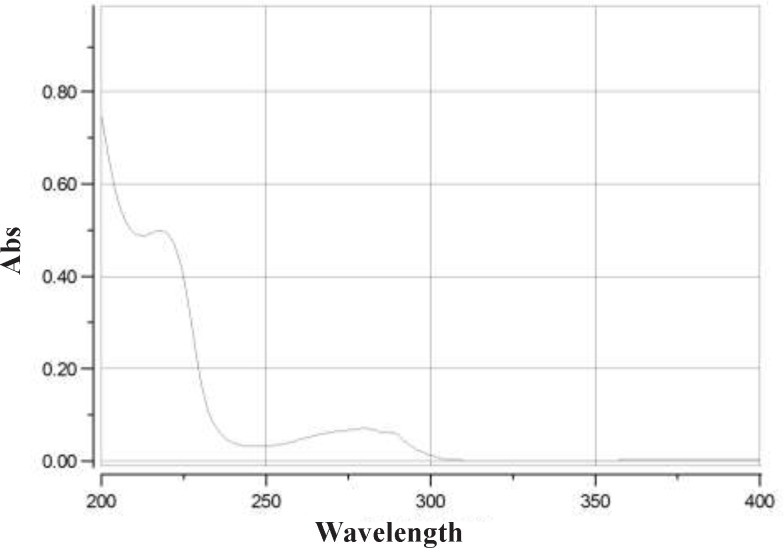
UV spectrum of eptifibatide acetate

**Figure 3:**
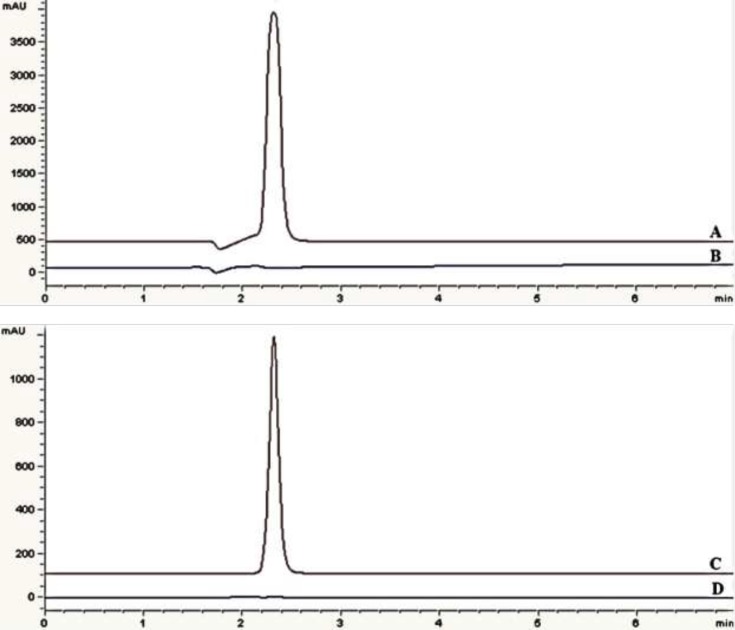
Chromatograms of: (A) standard solution, 0.75 mg/mL eptifibatide acetate in 219 nm (B) blank sample (deionized water) in 219 nm (C) standard solution, 0.75 mg/mL eptifibatide acetate in 275 nm (D) blank sample (deionized water) in 275 nm

**Figure 4:**
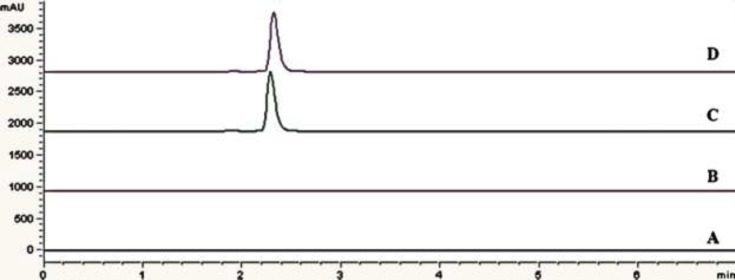
Chromatograms of: (A) blank sample (deionized water) (B) blank sample (citric acid buffer as excepient) (C) standard solution, 0.75 mg/mL eptifibatide acetate (D) Assay sample injection (Integrilin^® ^0.75 mg/mL

**Figure 5:**
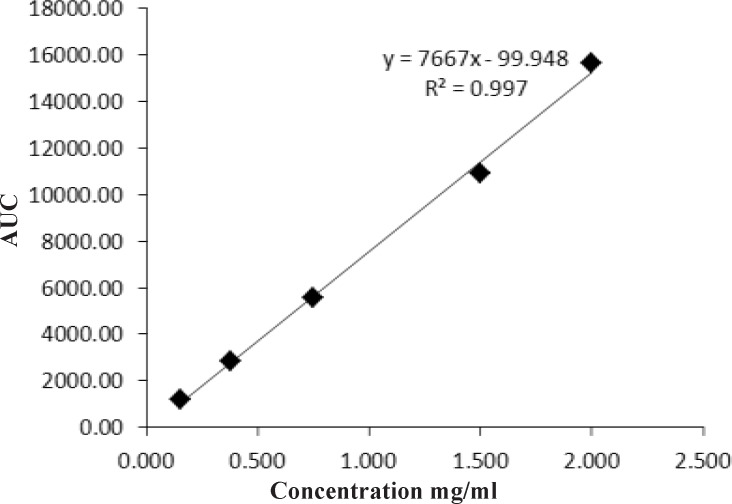
Linearity plot for eptifibatide drug substance.

**Table 1:** Intra-day and inter-day precision and accuracy of eptifibatide QC samples using the described HPLC method.

**Theoretical concentration**	**Calculated concentration** **(mean ± S.D., n = 3)**	**Precision** **(R.S.D.) (%)**	**Accuracy** **Recovery %**
**Intra-day**			
0.375	0.383 ± 0.001	0.053	102.11
0.750	0.783 ± 0.001	0.058	98.39
1.500	1.441 ± 0.001	0.052	96.07
**Inter-day**			
0.375	0.387 ± 0.002	0.598	103.18
0.750	0.740 ± 0.003	0.364	98.60
1.500	1.445 ± 0.003	0.204	96.36

**Table 2.** Experimental values of mean concentration, %RSD and %Recovery for stability studies of QC samples.

**Condition**	**Theoretical concentration**	**Calculated concentration** **(mean ± S.D., n = 3)**	**Precision** **(R.S.D.) (%)**	**Accuracy** **Recovery %**
Short term storage	0.375	0.385 ± 0.001	0.103	102.73
	0.750	0.737 ± 0.002	0.225	98.29
	1.500	1.472 ± 0.001	0.054	98.12
long term storage	0.375	0.387 ± 0.001	0.154	103.23
	0.750	0.400 ± 0.001	0.103	98.70
	1.500	1.549 ± 0.009	0.603	103.27
Freeze and thaw	0.375	0.384 ± 0.001	0.175	102.53
	0.750	0.739 ± 0.001	0.148	98.58
	1.500	1.511 ± 0.011	0.742	100.74

Eptifibatide acetate is a white or white-off powder, soluble in water and freely soluble in 1% acetic acid in water. Its empirical formula is C_35_H_49_N_11_O_9_S_2_ with molecular weight of 831.96.

From the literature survey, it was revealed that few spectrometric and chromatographic analytical methods have been developed for determination of eptifibatide in pharmaceutical preparations and biological fluids (7-11). Characterization of eptifibatide impurities during stability assays by using mass spectrometers coupled with a reverse phase gradient HPLC system has been performed by Wang *et al.* in 2003. Zhao *et al*. reported an isocratic RP-HPLC method for the assay of eptifibatide during drug stabilization studies (12). 

In this method, quantification of eptifibatide was achieved with UV detection at 220 nm (RT: 10 min). Kota *et al.* and Saksena *et al.* developed different reverse phase gradient HPLC methods for purity checking of synthetic eptifibatide substance after synthesis and purification procedures, but validation and applicability of these methods for marketed formulations were not reported (13, 14). Kota *et al.* used ACN and water as mobile phase. In this method, eptifibatide analysis time was 20 min. Saksena *et al.* presented method using ethanol and water as mobile phase (RT: 30 min). The drawbacks of the reported methods were the need for using gradient LC separation method with long run time and use of mass spectrometry that might not be universally available in laboratories due to its cost implications. 

Eptifibatide acetate has been approved by FDA 1998. However no compendia method has been published for its determination in bulk and pharmaceutical dosage forms. Considering the less complication and readily availability of HPLC-UV detection, the main objective of present study was to develop a rapid, economical and validated method for the assay and quality control of eptifibatide acetate in bulk drug substance and finished pharmaceutical dosage forms (15-21). The validation of chromatographic parameters was performed in accordance with ICH guidelines (22).

*Chemicals and reagents*

The tested pharmaceutical dosage forms (Integrilin^®^) were purchased from Merck and Co., Inc., (New Jersey, USA). Eptifibatide acetate working standard were purchased from HangZhou Think Chemical Co., Ltd. (HangZhou, China). Acetonitrile, methanol and TFA were of HPLC grade from Merck (Hohenbrunn, Germany). HPLC grade water was prepared using a Milli-Q purification system. All other reagents were of analytical grade.

*Instrumentation*

The LC system used for method development and validation consisted of an Agilent 1200 Series HPLC System equipped with autosampler (G1329A), UV detector (G1314B), degasser (G1379B), and binary pump (G1312A) (GenTech Scientific, NY, USA). Data acquisition, analysis and reporting were performed by ChemStation Software Rev.B.03.01.

*Preparation of stock and working standard solutions*

The stock standard solution of eptifibatide was prepared in deionized water at 10 mg/mL concentration. Then five working standard solutions (2, 1.5, 0.75, 0.375 and 0.15 mg/mL) were prepared by serial dilutions of the stock solution in deionized water. All the standard stock and working solutions were daily prepared.

*Preparation of quality control solutions*

The quality control (QC) stock solution of eptifibatide independent of the standard stock solution was prepared in deionized water. Then the QC sample solutions (1.5, 0.75 and 0.375 mg/mL) were prepared by serial dilutions of the QC stock solution in deionized water. All the QC stock and sample solutions were daily prepared.

*Selection of wavelength*

Determination of detection wavelength was based on the scanned UV spectrum of the eptifibatide acetate solution over the range of 200 to 400 nm. Maximum absorbance was observed at 219 and 275 nm.

*Chromatographic conditions*

The chromatographic separation was performed using isocratic elution at ambient temperature (25 °C) on Lichrospher^® ^C18 column (150 x 4.60 mm i.d., 5 µM particle size) with UV detection at 275 nm. The mobile phase was composed of the mixture of Solution A (0.1% (v/v) TFA in water) and Solution B (0.1% (v/v) TFA in Acetonitrile) at the ratio of 68:32 (v/v). The flow rate was set at 1 mL/min. The injection volume was 20 µL for every injection. 

*Analytical method validation*

The developed RP-HPLC method was validated in terms of the following parameters; specificity, linearity, sensitivity, precision, accuracy and stability of analytical solutions. The validation was carried out according to International Conference on Harmonization (ICH) guidelines for validation of analytical procedures (22).

*Specificity*

The specificity and selectivity of method was investigated by injecting of blank samples (deionized water and citric acid buffer as excipient) to demonstrate the absence of interference with elution of eptifibatide in standard samples or pharmaceutical formulation.

*Linearity*

To study the linearity of analytical procedure, five working standard solutions at different concentration levels (0.15-2 mg/mL) were injected into HPLC system in three individually replicates. In order to determine that the instrumental response was directly proportional to analyte concentration, calibration curve was constructed by plotting concentration of eptifibatide on X-axis and average peak area on Y-axis. Regression equation and value of co-relation coefficient was calculated using linear regression analysis.

*Sensitivity*

The sensitivity of the analytical technique was estimated in terms of limit of detection (LOD) and limit of quantification (LOQ). LOD and LOQ were defined based on signal to noise ratio of 3:1 and 10:1, respectively. LOQ was taken as lowest concentration of eptifibatetide that could be quantitatively determined with acceptable accuracy and precision (22).

*Precision and accuracy*

QC samples at three different concentration levels (0.375, 0.75 and 1.5 mg/mL) were analyzed during three consecutive days (inter-day precision) and three times during the same day (intra-day precision). The precision of proposed method was obtained by calculating the relative standard deviation (RSD) values for intra-day and inter-day analysis with acceptance criteria of not more than 2% (22). The accuracy of measurement method was assessed via the methodological recovery. The percentage deviation of determined concentration of QC sample and theoretical concentration expressed the recovery of method (22). 

*Stability of analytical solutions*

To evaluate stability of samples during the analysis, stability of QC samples under 3 different conditions were checked by replicated analysis (N = 3). After short-term storage (at 25 °C for 24 h), after long-term storage (at 2-8 °C for 2 weeks) and after going through three freeze-and-thaw cycles (from -20 to room temperature for every 24 h), aged QC samples reanalyzed versuse freshly prepared standard solution.

*Assay of marketed formulation*

To define applicability of assay method to commercial formulation, 10 vials of solution for injection (Integrilin^®^ 2 mg/mL) and solution for infusion (Integrilin^®^ 0.75 mg/mL) were analyzed, respectively. Quantification of injections was achieved using the regression equation.

## Results and Discussion

*Selection of wavelength*

UV spectrum of eptifibatide acetate solution showed maximum absorbance at 219 and 275 nm (Figure 2). The assay chromatograms at 219 and 275 nm are presented in Figure 3. The wavelength of 275 nm was selected as suitable detection wavelength because of clear flat baseline and being prevented from interference coming from TFA along with symmetrical response peak.

*HPLC method development*

The present work was aimed at developing simple, rapid and economical assay method for eptifibatide acetate in API powder and dosage forms. The RP-HPLC separation was developed on C18 column under isocratic condition with short retention time (<3 min), acceptable resolution, use of cost-effective solvents and ease of preparation. Quantitative analysis was achieved with high chromatographic response peak and optimum resolution.

*Specificity*

Figure 4 shows overlay of chromatograms for standard sample and assay sample (solution for injection) with that of blank samples (deionized water and citric acid buffer as excipient). No interference with eptifibatide peak was observed as blank has no interfering peak at retention time of eptifibatide. Specificity of the method can be concluded based on the chromatographic peak purity observed in the chromatograms (22).

*Linearity*

The calibration curve was constructed by plotting a graph of peak mean area versus concentration. The linearity of calibration curve was evaluated using linear regression analysis. Regression equation was: Y = 7667X- 99.948. Co-relation coefficient was 0.997, which meet the analytical method validation acceptance criteria (22). Hence linearity of method was proved over the concentration of 0.15-2 mg/mL (Figure 5).

*Sensitivity*

LOD and LOQ of the method were calculated, using signal to noise ratio method, 15 and 45 µg/mL, respectively. However the recovery of quantification at LOQ concentration level was 70%, which does not meet the acceptance criteria for method recovery. 

Therefor the lowest concentration on calibration curve that can be reproducibly quantified with acceptable accuracy and precision, 0.15 mg/mL, was experimentally considered as LOQ of the assay method (22).

*Precision*

The intra-day and inter-day precision of method was expressed as RSD value. The RSD values for intra- and inter-day assay of QC samples did not exceed 2%, thus indicating the good precision of the method. The results are presented in Table 1.

*Accuracy*

The accuracy of method was assessed by recovery studies on QC samples. The percent recovery by the assay of QC samples ranged from 96.36 to 103.18%. The good recovery values for accuracy study ascertain that method is accurate as shown in Table 1.

*Stability of analytical solution*

Stability results obtained from stability studies of QC samples stored in different conditions are summarized in Table 2. Based on these results, QC samples are considered stable for 24 h at room temperature, up to 14 days when stored under refrigeration and after three freeze-and-thaw cycles.

*Application of the method for the analysis of Eptifibatide acetate injection formulation (Integrilin* ^®^ *)*

The validated developed method was applied for the analysis of eptifibatide acetate injection forms (Integrilin^®^ 0.75 mg/mL and 2 mg/mL). The results of average assays (N = 10) yield 103.446 ± 0.001 and 100.100 ± 0.003 for solution for injection and solution for infusion formulation, respectively. Short analysis time and low RSD value with acceptable accuracy indicate suitability of this method for routine analysis of eptifibatide marketed injection forms.

## Conclusion

A simple, rapid, validated and isocratic RP-HPLC method with UV detection was developed for the identification and quantification of eptifibatide acetate in pure and pharmaceutical formulation. The method was successfully validated as per ICH guidelines and statistical data confirmed selectivity, linearity, sensitivity, precision and accuracy of proposed method. Also, the described method can be used to stability study of analytical solutions. Because of importance of short retention time in routine analysis of drug, the current method sounds to be applicable as quality control tool for assay of eptifibatide acetate in pharmaceutical industries. 
